# Supplementary figures and images for: Isolation, Identification and Hyperparasitism of a Novel Cladosporium cladosporioides Isolate Hyperparasitic to Puccinia striiformis f. sp. tritici, the Wheat Stripe Rust Pathogen
Source: Biology (Basel). 2022 Jun 10;11(6):892. doi: 10.3390/biology11060892 (PMC9219750; doi:10.3390/biology11060892)

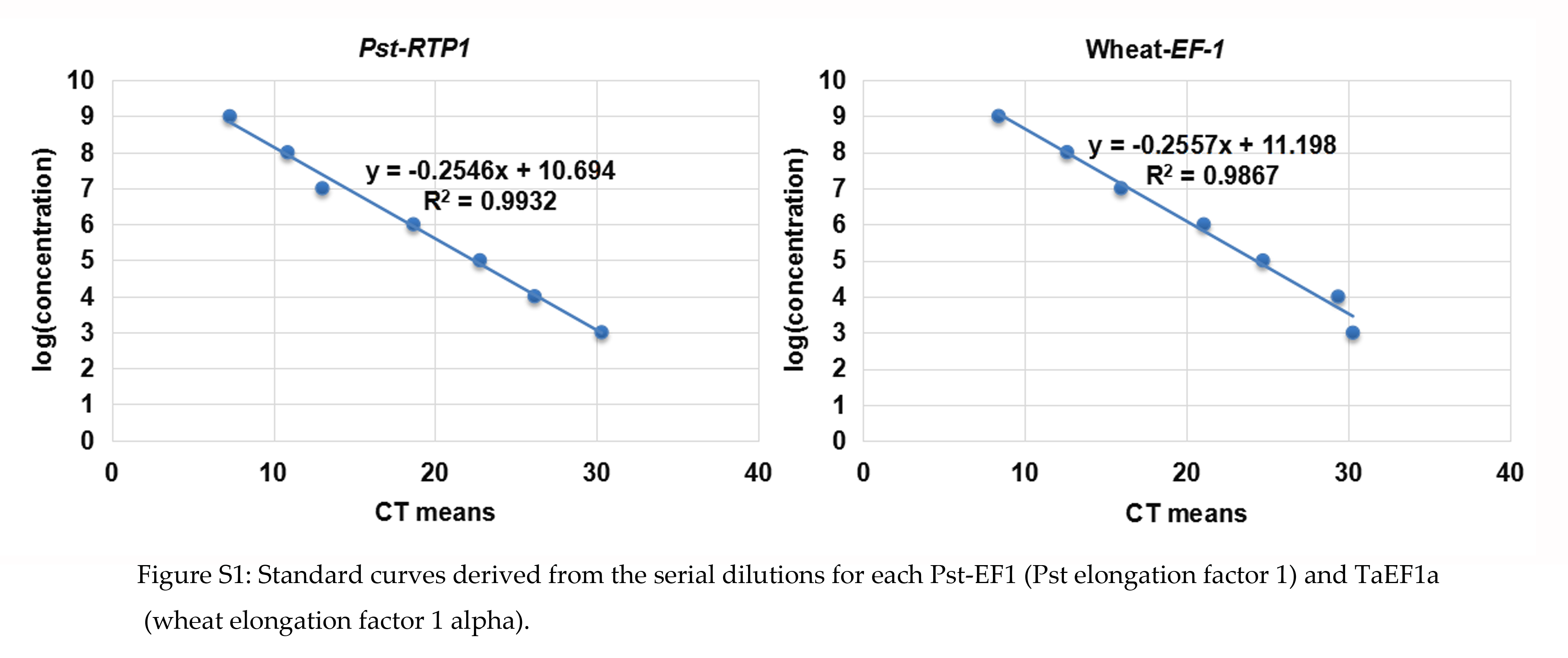

Supplement: Supplementary file 1 [file biology-11-00892-s001.zip › biology-1722403-Figure S1.tif]
